# Associations of serum levels of microRNA-371a-3p (M371) with risk factors for progression in nonseminomatous testicular germ cell tumours clinical stage 1

**DOI:** 10.1007/s00345-021-03876-2

**Published:** 2021-11-14

**Authors:** Klaus-Peter Dieckmann, Cansu Dumlupinar, Arlo Radtke, Cord Matthies, Renate Pichler, Pia Paffenholz, Jörg Sommer, Alexander Winter, Friedemann Zengerling, Finja Hennig, Christian Wülfing, Gazanfer Belge

**Affiliations:** 1 Department of Urology, Asklepios Klinik Altona, Hamburg, Germany; 2 Faculty of Biology and Chemistry, University Bremen, Leobener Strasse 2/FVG, 28359 Bremen, Germany; 3 Mirdetect GmbH, Bremerhaven, Germany; 4 Department of Urology, Bundeswehrkrankenhaus Hamburg, Hamburg, Germany; 5 Department of Urology, University Hospital Innsbruck, Innsbruck, Austria; 6 Department of Urology, University Hospital Cologne, Cologne, Germany; 7 Department of Urology, St. Franziskus Krankenhaus Lohne, Lohne, Germany; 8 Department of Urology, University Hospital Oldenburg, Oldenburg, Germany; 9 Department of Urology, University Hospital Ulm, Ulm, Germany

**Keywords:** Germ cell tumour, Tumour marker, MicroRNA, Vascular invasion, Embryonal carcinoma, Nonseminoma

## Abstract

**Purpose:**

Lymphovascular invasion (LV1) and presence of > 50% embryonal carcinoma (> 50% EC) represent risk factors for progression in patients with clinical stage 1 (CS1) nonseminomatous (NS) testicular germ cell tumours. As serum levels of microRNA-371a-3p (M371) are capable of detecting small amounts of GCT, we evaluated if LV1 and > 50% EC are associated with M371 levels.

**Methods:**

M371 serum levels were measured postoperatively in 153 NS CS1 patients and both pre- and postoperatively in 131 patients. We registered the following factors: age, tumour size, LV status, > 50% EC, teratoma in primary, preoperative elevation of classical tumour markers. M371 expression was compared among subgroups. The ability of M371 to predict LV1 was calculated by receiver operating characteristics (ROC) curves. Multiple regression analysis was used to look for associations of M371 levels with other factors.

**Results:**

Postoperatively elevated M371 levels were found in 29.4% of the patients, but were neither associated with LV status nor with > 50% EC. Likewise, relative decrease of M371 was not associated. ROC analysis of postoperative M371 levels revealed an AUC of 0.5 for the ability to predict LV1 while preoperative M371 had an AUC of 0.732. Multiple regression analysis revealed significant associations of preoperative M371 levels with LV status (*p* = 0.003), tumour size (*p* = 0.001), > 50% EC (*p* = 0.004), and teratoma component (*p* = 0.045).

**Conclusion:**

Postoperatively elevated M371 levels are not associated with risk factors for progression in NS CS1 patients. However, the significant association of preoperative M371 expression with LV1 deserves further evaluation.

## Introduction

Clinical stage 1 (CS1) nonseminomatous tumours (NS) comprise of 20% of all patients with testicular germ cell tumours (GCTs) [1]. However, accurate clinical staging is hampered by the inability of imaging procedures to detect micro-metastatic seeds [2, 3] and by the non-expression of tumour markers alpha fetoprotein (AFP), beta human chorionic gonadotropin (bHCG) and lactate dehydrogenase (LDH) in almost 50% of the cases [4, 5]. Approximately, 30% of NS cases classified as clinical stage 1 (CS1) do actually harbour microscopic neoplastic foci in the retroperitoneal nodes that will inevitably progress to overt metastatic disease [6]. Clinico-pathological risk factors may predict the presence of occult metastases and may, thus, aid decision-making with respect to prophylactic chemotherapy [7, 8]. Lymphovascular invasion (LV1, also characterised as pathological stage pT2 according to the TNM classification) is the most widely recognised risk factor that indicates a 50–60% risk of developing metastases if no treatment is administered [9]. Another promising risk marker is the presence of embryonal carcinoma (EC) in the primary tumour [10]. However, it is unresolved which proportion of EC is relevant for predicting occult metastases [11]. Other factors, e.g. tumour size and stromal rete testis invasion have shown some promise but have not yet reached international consensus. Noteworthy, lymphovascular invasion involves low specificity, since 15–20% of CS1 NS patients will progress despite the absence of vascular invasion (LVo) and conversely, about 40–50% with the factor (LV1) will not. Another problem of histology-based risk factors is inter-observer variation in the assessment of testicular pathological details which relates to the over-all rarity of testicular neoplasms [12, 13].

Serum levels of microRNA-371a-3p (M371) have been shown to be a promising novel biomarker of germ cell tumours outperforming the classical markers [14–16]. Even small GCTs of less than 10 mm of size can generate elevated M371 serum levels [17, 18]. Based on the high sensitivity of > 90%, it has been speculated that this marker would also be informative in patients harbouring small volume occult metastases [19, 20]. A prospective study had shown that M371 serum levels dropped after orchiectomy in 92% of CS1 patients, but around 20% of them had still elevated M371 levels postoperatively [17]. Although no follow-up of these particular cases was available, the hypothesis was raised that elevated M371 levels after surgery of CS1 patients could denote those with occult metastases [16]. If this hypothesis is correct, then a large number of patients with postoperatively elevated M371 levels are expected to have the hitherto known predictors of progression in their primary tumours. At present, there are no data to substantiate this possible association. We measured M371 serum levels in patients with CS1 testicular NS and tested the following hypotheses: (1) both, LV1 and predominance of EC are associated with postoperatively elevated M371 levels, (2) preoperative M371 levels are higher in patients with risk factors LV1 and/or EC than in those without these factors, and (3) the relative decrease of postoperative M371 levels is greater in patients with the factor (LV1) than in those without (LVo). In addition, we looked for associations of a number of other clinico-pathological factors with M371 levels in an exploratory approach.

## Patients, methods

In a multicentric study, a total of 153 patients with CS1 NS testicular tumours aged 18–56 years, underwent measurement of postoperative serum levels of M371 of whom 131 also had measurements before orchiectomy. After blood aspiration serum aliquots were kept deep-frozen at minus 80 °C until processing. The laboratory measurement technique had been fully described earlier [17]. A serum level of RQ = 5 was considered the upper limit of norm (ULN). Patients were prospectively enrolled during 2016–2020, and 81 had been included in previous evaluations [17, 21]. In each case we registered patient´s age (years), presence of lymphatic or vascular invasion of the tumour (LV1: yes/LVo: no), presence of > 50% EC in the primary (yes/no), teratoma as component of the primary (yes/no), tumour size (mm), preoperative elevation of AFP, bHCG, and LDH, respectively (yes/no). Histopathological details were retrieved from local pathology reports without central pathological review.

Patients´ data were initially stored in a commercially available data base system (MS Excel, Microsoft Corp., Redmond, USA, version 2017) and transferred to SPSS software (SPSS Inc., IBM Corp, Armonk, NY, USA, version 24) for final evaluation. Statistical analysis comprised of calculating median and interquartile ranges (IQRs) with regard to age and tumour size, respectively. We calculated relative frequencies of the presence of LV1, > 50% EC, teratoma as component, and elevations of classical tumour markers. Median and IQRs of the relative quantity (RQ) values of preoperative and postoperative M371 serum levels were calculated. To look for associations of M371 expression with vascular invasion, we compared the subgroups of LV1 and LVo with regard to the frequencies of M371 elevations and also regarding the median RQ values of serum levels of the two subgroups. Separate comparisons were done with preoperative and postoperative measurements. The same calculations were done with the subgroups with > 50% EC and < 50% EC, respectively. The subgroups of LV1 and LVo were compared to each other with regard to the absolute and relative decreases from preoperative M371 to postoperative levels. Receiver operating characteristics curves (ROC) were calculated to evaluate the sensitivity and specificity of preoperative and postoperative M371 expression to predict LV status. We also compared the subgroups LV1 and LVo with regard to frequencies of M371 elevations in marker-positive and marker-negative patients.

For statistical comparison of proportions (univariate categorical variables), the chi-squared test was used. For comparison of continuous variables, the Mann–Whitney *U* test and Wilcoxon signed-rank test were used. A *p* < 0.05 was considered significant. The association of preoperative M371 expression with tumour size in either LVo or LV1 was determined with linear regression analysis. Multiple regression analysis was used to look for the dependence of M371 expression from the factors tumour size, patients’ age, EC content, classical marker expression and the LV status.

The study received ethical approval by Ärztekammer Bremen (#301, 2015). All patients gave informed consent prior to their inclusion in the study. All of the study activities conformed to the Helsinki Declaration of the World Medical Association (as amended by the 64th General Assembly, 2013).

## Results

Elevated M371 levels were found in 111 patients before orchiectomy (84.7%), and postoperatively in 45 patients (29.4%). Lymphovascular invasion (LV1) was registered in 64 patients (41.8%). Further clinical and pathological details of the patients are listed in Table 1.

**Table 1:** Patients’ characteristics

	Eligible (*n*)	Results
Patients’ age: median; IQR [years]	153	30; 25.5–37.0
M371—preoperative serum level elevated (*n*, %)	131	111 (84.7%)
M371—postoperative serum level elevated (*n*, %)	153	45 (29.4%)
With vascular invasion (LV1) (*n*, %)	64	64 (41.8%)
Without vascular invasion (LVo) (*n*, %)	89	89 (58.2%)
With > 50% EC in primary tumour (*n*, %)	152	79 (52.0%)
Teratoma component in primary tumour (*n*, %)	152	83 (54.6%)
Tumour size: median; IQR [mm]	149	27; 18.0–42.0
AFP—preoperative serum level elevated (*n*, %)	152	75 (49.3%)
bHCG—preoperative serum level elevated (*n*, %)	152	66 (43.4%)
LDH—preoperative serum level elevated (*n*, %)	147	13 (8.8%)
Any marker elevation preoperatively (*n*, %)	149	93 (62.4%)

*LV1* lymphovascular invasion, *LVo* without lymphovascular invasion, *EC* embryonal carcinoma, *IQR* interquartile range

**Fig. 1:**
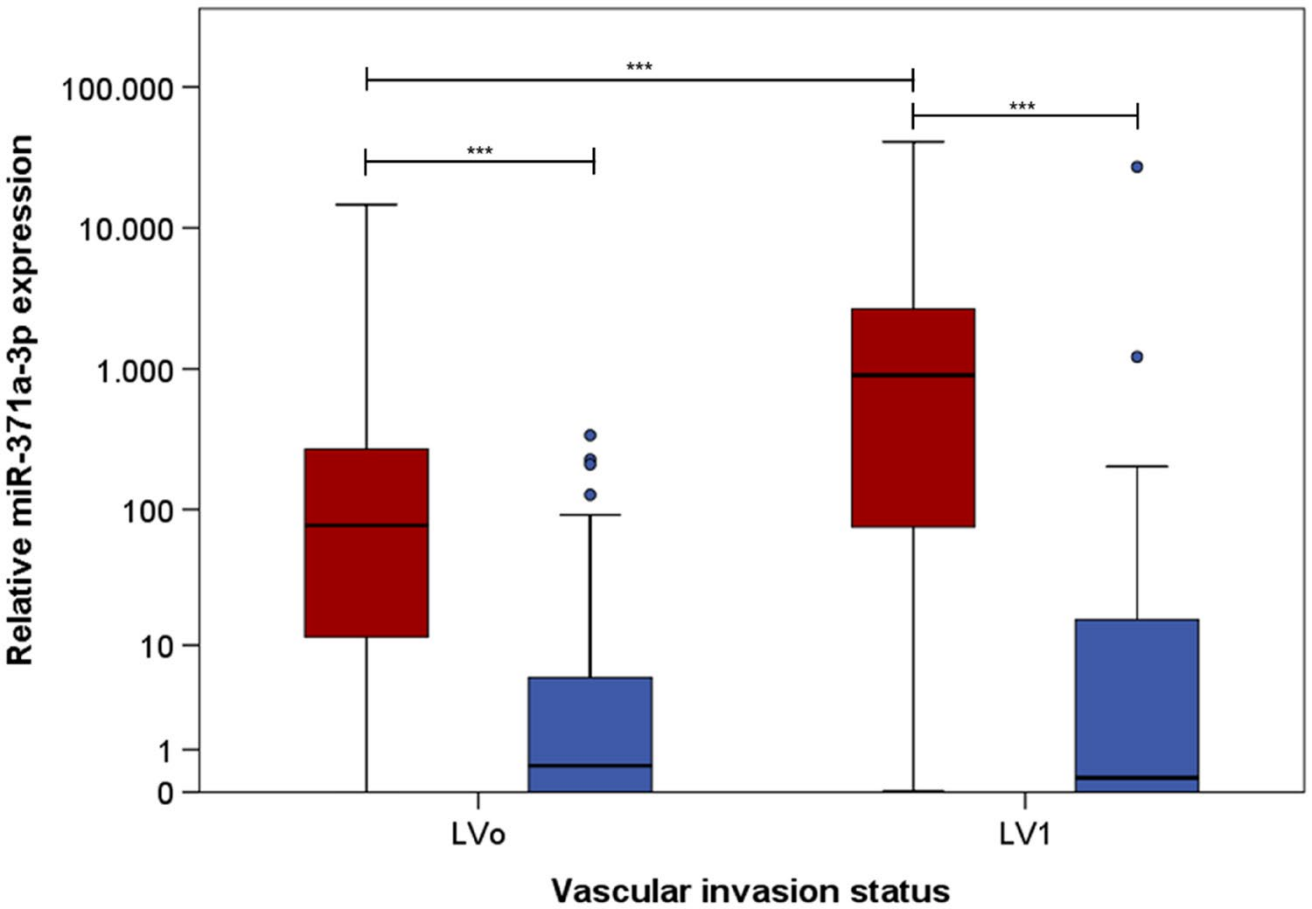
M371 serum levels in patients with and without lymphovascular invasion. Box plots of the relative M371 expression in patients with lymphovascular invasion in primary tumour (LV1; *n* = 64) and without (LVo; *n* = 89). Comparison of preoperative median levels (red) with postoperative levels (blue). The *y* axis is plotted in a logarithmic scale. Horizontal bars highlight the significant statistical comparisons. All differences are significant (***p < 0.001)

**Fig. 2:**
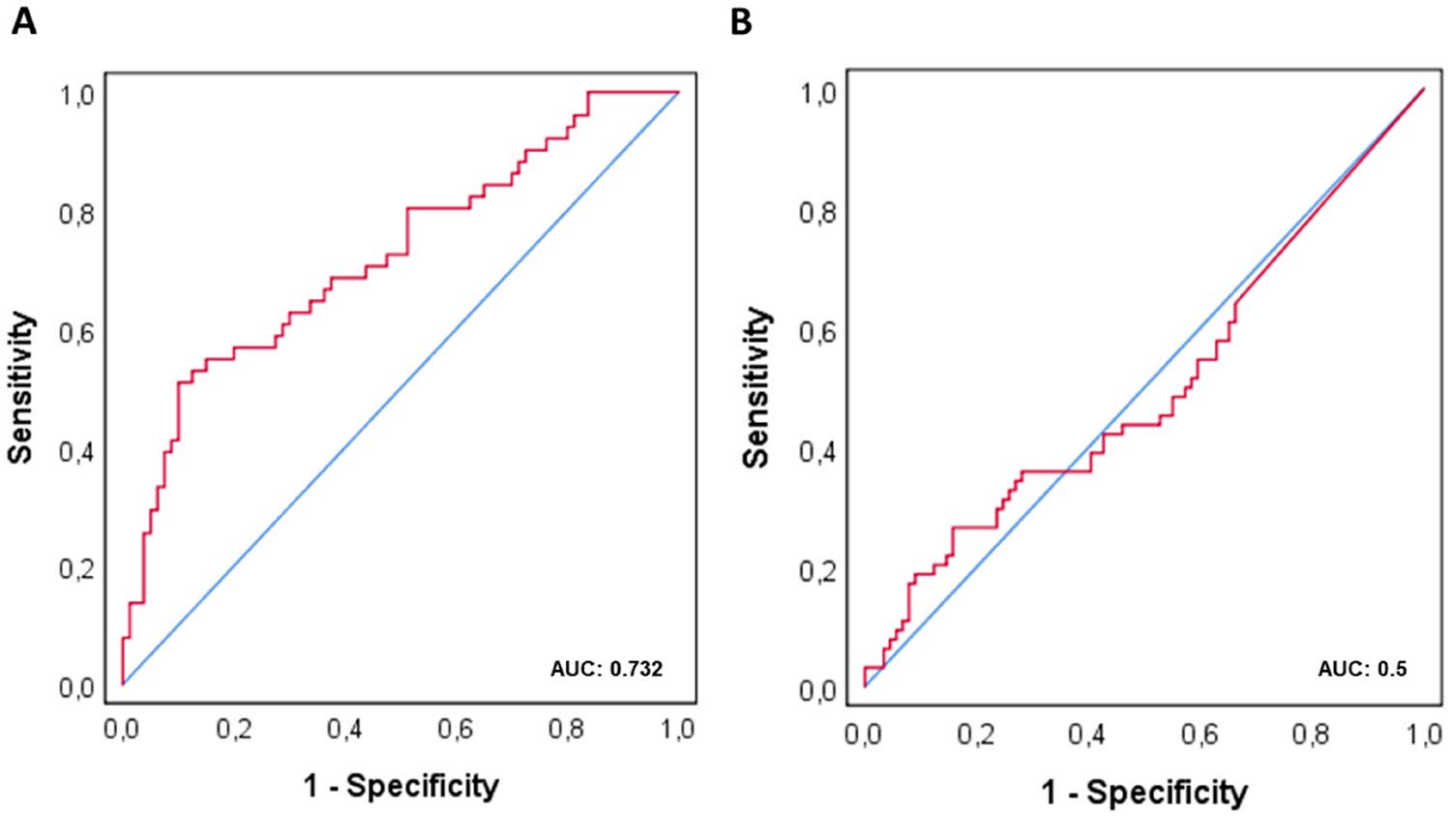
**A** Ability of preoperative M371 serum level to predict lymphovascular invasion. Receiver operating characteristics curve (ROC) showing the ability of preoperative M371 levels to discriminate between LVo and LV1 status. This analysis involved 51 patients with LV1 and 80 with LVo; the AUC is 0.732. **B** Ability of postoperative M371 serum level to predict lymphovascular invasion. ROC curve showing the ability of postoperative M371 levels to discriminate between LVo and LV1 status. This analysis involved 64 patients with LV1 and 89 with LVo. The AUC is 0.5

The preoperatively measured M371 serum levels are significantly higher in the LV1 subgroup than in the LVo group. The postoperative median M371 level of the LV1 subgroup is not different from that of the LVo subgroup (Table 2, Fig. 1). ROC curves revealed an AUC of 0.732 for the ability of preoperative M371 levels to predict the LV1 status (Fig. 2a). Postoperative M371 levels are not associated with the LV status as shown by an AUC of 0.5 in the ROC curve (Fig. 2b).

**Table 2:** Results of preoperative and postoperative of M371 measurements and comparisons of subgroups LVo versus LV1

	Eligible (*n*)		LVo	LV1	*p* value	Comparison LVo vs LV1
Preoperative M371 measurement (*n*)	131		80	51		
M371 elevated (*n*; %)	111	84.7%	64 (80.0%)	47 (92.2%)	0.081	Proportions of M371 elevation^a^
M371 elevated (median RQ; IQR)			76.74; 11.51–271.78	902.13; 74.70–2724.65	0.000012	Comparison of RQ values^b^
Postoperative M371 measurement	153		89	64		
M371 elevated (*n*; %)	45	29.4%	24 (27.0%)	21 (32.8%)	0.475	Proportions of M371 elevation^a^
M371 elevated (median RQ; IQR)			0.54; 0.00–5.49	0.26; 0.00–15.99	0.995	Comparison of RQ values^b^

*RQ* relative quantity, *IQR* interquartile range, *LV1* lymphovascular invasion, *LVo* without lymphovascular invasion

^a^Chi-squared test

^b^Mann–Whitney *U* test

**Fig. 3:**
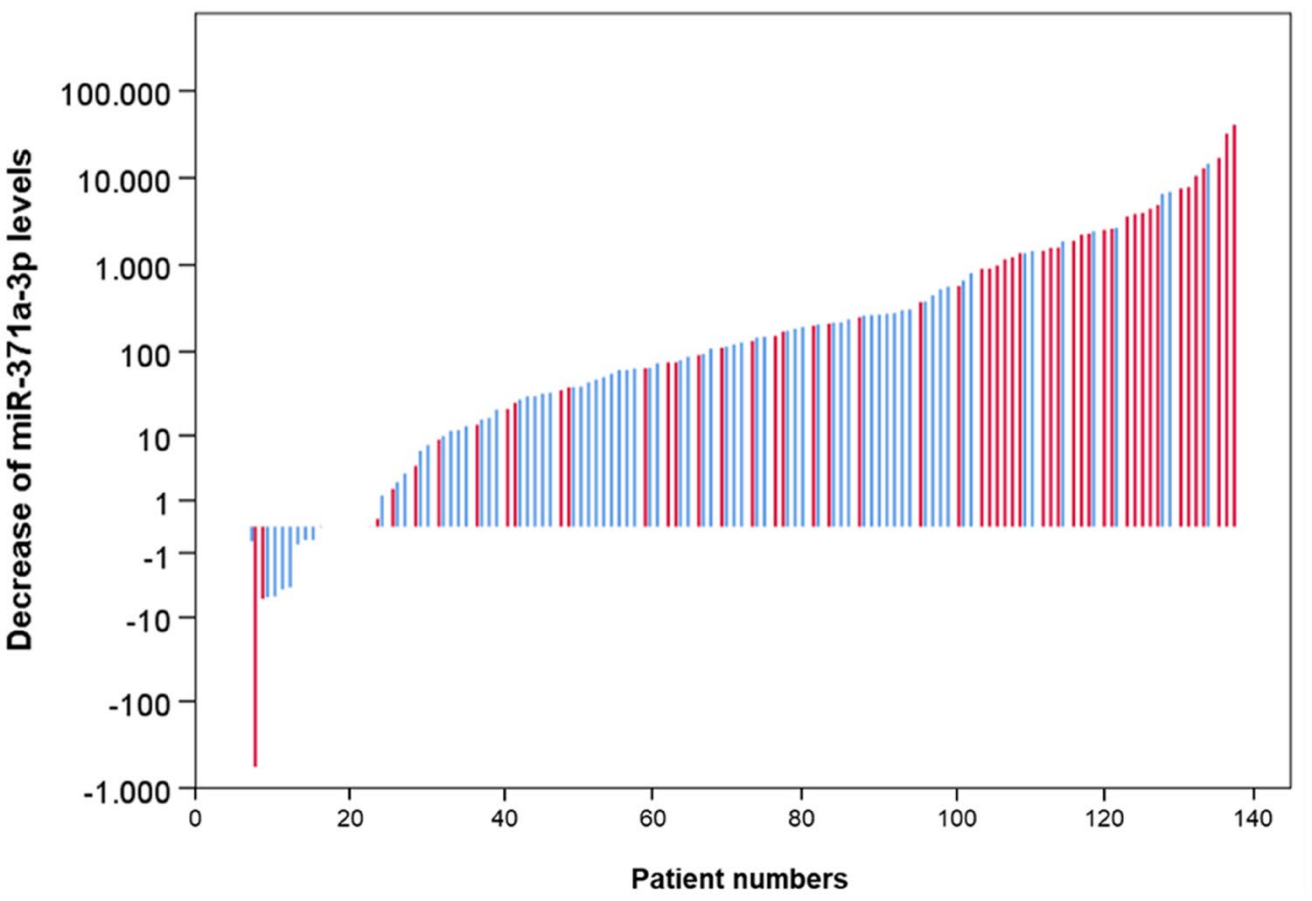
Decreases of M371 serum levels after orchiectomy in individual patients with and without lymphovascular invasion. Waterfall plot showing absolute decreases of M371 levels following orchiectomy in 131 individual patients with paired measurements before and after orchiectomy, thereof 80 LVo patients (blue), and 51 LV1 patients (red). Each vertical bar represents one individual patient. The LV1 subgroup involves significantly greater declines than the LVo subgroup (*p* = 0.000059). The *y* axis is displayed in a logarithmic scale

The relative decrease of serum M371 levels after surgery is not significantly different among the subgroups of LV1 and LVo. However, if absolute differences of RQ levels (preoperative M371 minus postoperative M371 level) are analysed in a waterfall plot (Fig. 3), it becomes obvious that LV1 patients have significantly greater decreases of M371 after surgery than LVo patients (*p* = 0.000059, Mann–Whitney *U* test).

Regarding embryonal carcinoma, the preoperative median M371 level of the > 50% EC subgroup is significantly higher than that of the subgroup with < 50% EC (*p* = 0.008, Mann–Whitney *U* test; Table 3). Also, the proportion of elevated M371 levels is significantly higher in the > 50% EC group (93.9%) than in the group with < 50% EC (75.4%) (*p* = 0.003). Postoperatively, the > 50% EC subgroup had more frequently elevated levels than the < 50% EC subgroup (35.4% versus 23.3%), however that difference is not significant (*p* = 0.112). The median postoperative RQ value is higher in the > 50% EC group than in the < 50% EC group (1.3 versus 0.23, *p* = 0.061); however, both values are below ULN (RQ = 5).

**Table 3:** Impact of presence of embryonal carcinoma in the primary on M371 levels: preoperative and postoperative of M371 measurements and comparisons of subgroups

	Eligible (*n*)	< 50% EC	> 50% EC	*p* value	Comparison < 50% EC vs. > 50% EC
Preoperative M371 measurement (*n*)	131	65	66		
M371 elevated (*n*; %)	111 (84.7%)	49 (75.4%)	62 (93.9%)	0.003	Proportions of M371 elevation^a^
M371 elevated (RQ median; IQR)		63.1; 5.28–853.31	225.0; 67.24–1208.68	0.008	Comparison of RQ values^b^
Postoperative M371 measurement	152	73	79		
M371 elevated (*n*; %)	43 (28.9%)	17 (23.3%)	28 (35.4%)	0.112	Proportions of M371 elevation^a^
M371 elevated (RQ median; IQR)		0.23; 0.00–4.36	1.3; 0.00–17.19	0.061	Comparison of RQ values^b^

*EC* embryonal carcinoma, *IQR* interquartile range, *RQ* relative quantity

^a^Chi-squared test

^b^Mann–Whitney *U* test

The elevation of classical tumour markers was not associated with preoperative M371 expression both in LV1 and LVo patients (Table 4). In marker-negative patients, the subgroups of LV1 and LVo revealed frequencies of M371 expression in 83.3% and 71.4%, respectively, while in marker-positive patients the frequencies were 96.7% and 84.3%, respectively. The differences between LV1 and LVo were not significant in both comparisons.

**Table 4:** Comparison of subgroups LVo versus LV1 with regard to preoperative M371 expression in patients with and without elevation of classical markers (“marker-negative” and “marker-positive” patients)

	Eligible (*n*)	LVo	LV1	*p* value	Type of comparison
Marker-negative	46				
With M371 expression (*n*; %)	35	20 (71.4%)	15 (83.3%)		
No M371 expression (*n*; %)	11	8 (28.6%)	3 (16.7%)	0.486	Comparison of proportions^a^
Marker-positive	81				
With M371 expression (*n*; %)	72	43 (84.3%)	29 (96.7%)		
No M371 expression (*n*; %)	9	8 (15.7%)	1 (3.3%)	0.143	Comparison of proportions^a^

*LV1* lymphovascular invasion, *LVo* without lymphovascular invasion

^a^Chi-squared test

**Fig. 4:**
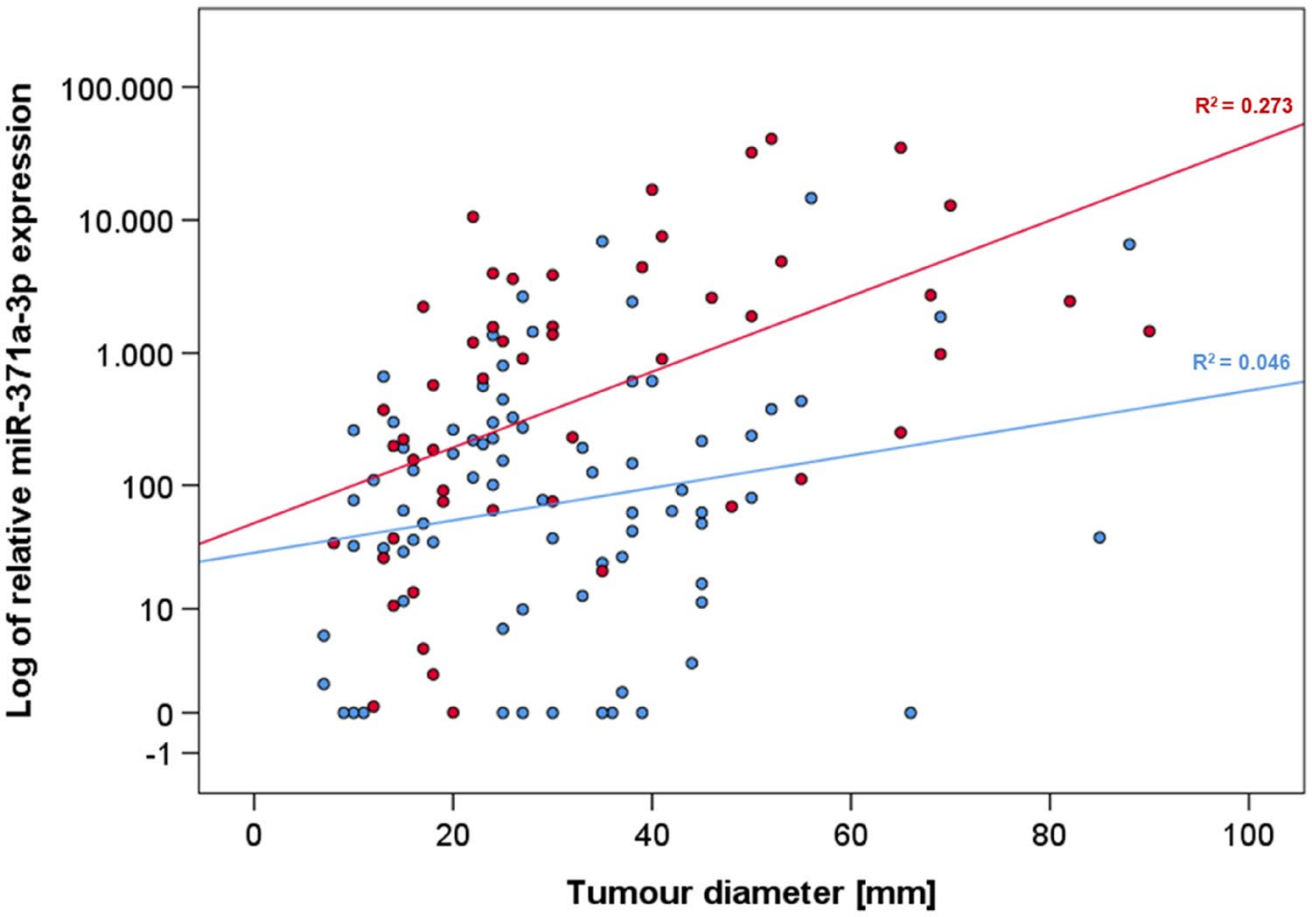
Association of preoperative M371 levels with tumour size in patients with and without lymphovascular invasion. Scatter plot with regression lines showing the association between the preoperative M371 levels and tumour diameter in individual patients. The association in LV1 patients (red) is higher with *R*^2^ = 0.273 (*p* = 0.0035) than in LVo patients (blue) *R*^2^ = 0.046 (*p* = 0.0089). The *y* axis is plotted in a logarithmic scale

Preoperative M371 levels are associated with tumour size as revealed by linear regression analysis (scatter plot, Fig. 4). Regression curves show that the association is greater in LV1 patients than in LVo patients with a much greater coefficient of variation in the LV1 patients (*R*^2^ = 0.273) than in those with LVo (*R*^2^ = 0.046). In both subgroups the slopes of the regression lines are significantly different from zero (*p* = 0.0035 and *p* = 0.0089, respectively), thus providing evidence for the association of the M371 expression with tumour size in both subgroups, LVo and LV1, with a stronger association in LV1.

Multiple regression analysis showed > 50% EC (*p* = 0.004), tumour size (*p* = 0.001), teratoma components (*p* = 0.045), and LV status (*p* = 0.003) to be significant predictors of preoperative miRNA expression (*p* = 0.000014; *R*^2^ = 0.239) but not of postoperative expression (*p* = 0.063; *R*^2^ = 0.083) (Table 5).

**Table 5:** Potential associations of preoperative and postoperative M371 serum levels elevations with clinico-pathological factors: multiple regression analysis

	Preoperative measurements *p* value	Postoperative measurements *p* value
Whole model	0.000014	0.063
Tumor size: > median; < median [mm]	0.001	0.019
Patient age: > median; < median [years]	0.335	0.056
EC in primary tumour [> 50%; < 50%]	0.004	0.105
Teratoma component in primary tumour [present; not present]	0.045	0.175
Classical markers [elevated; normal]	0.481	0.959
LV status (LVo; LV1)	0.003	0.245

Patients eligible: preoperatively *n* = 124; postoperatively *n* = 142

*EC* embryonal carcinoma, *LV1* lymphovascular invasion, *LVo* without lymphovascular invasion

## Discussion

Elevated M371 serum levels after orchiectomy were found in 29.4% of CS1 testicular NS patients which is consistent with previous reports [17, 20]. The clinically important question is whether persistently elevated M371 levels after surgery in CS1 patients involve any biological significance, particularly, if postoperative M371 levels > ULN relate to the presence of occult metastases and may, thus, indicate the probability of progression. The central result of the present study is that both, the presence of lymphovascular invasion (LV1) and the presence of > 50% embryonal carcinoma in the primary tumour are not associated with postoperative M371 elevation. Accordingly, almost identical postoperative M371 expression rates are found in LV1 and LVo patients, and the ROC analysis clearly shows that postoperative M371 levels can predict the LV1 status only with chance probability (AUC = 0.5).

The proportions of patients with decreasing M371 levels secondary to orchiectomy are not different among the two subgroups with risk factors (LV1; > 50% EC) and those without (LVo; < 50% EC), respectively. The extent of reduction from preoperative to postoperative M371 level is greater in the LV1 subgroup than in the LVo subgroup, when absolute RQ values are considered. However, this difference relates to comparatively higher preoperative RQ values in LV1 patients. Thus, the percent decline of M371 levels does probably not involve any information about the probability of progression.

Evidence for the clinical significance of postoperatively elevated M371 levels can only come from systematic follow-up observations of patients who do not receive adjuvant treatment. However, we do not have information about the later course of the patients of the present investigation because the original study design had only involved M371 measurements before and after surgery for exploring sensitivity and specificity of the test with no further clinical observation. Moreover, as treatment decisions were made by local physicians, many of the patients of the present multicentric study have received some sort of adjuvant treatment, preventing any meaningful conclusions about persisting M371 expression after orchiectomy. First data providing insight into the biological significance of postoperatively elevated M371 levels in CS1 NS patients came from a recent study from Lobo et al. who longitudinally examined banked sera of CS1 patients under surveillance without adjuvant therapy. Noteworthy, the postoperatively measured M371 levels of patients destined to relapse were not different from those who remained disease-free [16]. Also, the percent decline of M371 (from preoperative levels to postoperative) was not predictive for recurrence. These results are widely in accordance with our data although a direct comparison is clearly not possible because in our present study the LV1 status was used as surrogate marker for relapses.

Another way of evaluating the biological significance of postoperatively elevated M371 levels would be to look histologically for occult disease in the abdominal lymph nodes obtained by retroperitoneal lymph node dissection (RLND). However, this management of nonseminomatous GCT used to be the standard of care only some decades ago [22]. Presently, RPLND has lost its significance and surveillance is the preferred way of care in NS CS1 patients [7]. Yet recently, the Dallas group measured data on post-orchiectomy M371 levels in patients undergoing primary RPLND and found that the levels measured before RPLND accurately predicted the presence of viable GCT in resected nodes with an AUC of 0.965 [23]. However, that study involved only 24 patients and that small patient sample comprised of both CS1 and CS2 patients. Furthermore, no information was given with regard to the intervals from orchiectomy to the time point of postoperative M371 measurement. Thus, that study undoubtedly confirmed the ability of M371 to detect small neoplastic GCT foci but did not really provide a clue to the enigma of postoperatively elevated M371 levels in CS1 patients.

Thus far, no clearly defined biological role of postoperatively elevated M371 levels in CS1 NS patients has been elucidated, however, a hypothesis may be raised. As shown in this study and earlier reports [17, 24], there is a highly significant association of preoperative M371 levels with tumour size, and more generally, with tumour bulk [25, 26]. Another important observation is the very rapid decay of M371 serum levels with an estimated half-life of less than 24 h and an association of the velocity of decay with tumour size with larger tumours needing more time to drop below the ULN [27, 28]. One may, therefore, hypothesise that postoperatively elevated M371 levels may be found mainly in those patients with large tumours because in these cases, the decrease of M371 is less rapid.

Another hypothesis to explain persistent elevated M371 after surgery could be a too short time interval between surgery and blood sample acquisition. Despite the known short half-life of M371, blood sampling on the first postoperative day may reveal still elevated miR levels particularly in those with large primary tumours.

Clearly, the true biological significance of postoperatively elevated M371 levels can only be assessed in a study where all patients will have blood aspirations for M371 measurement done within a clearly defined time-frame, preferably not earlier than 5 days after orchiectomy. In this study, the day of blood sampling was not specified by the protocol and, therefore, ranged from days 1 to 21, postoperatively. In light of the very short hospital stays of testicular cancer patients, a considerable number of whom will have had their postoperative M371 measurements at times when the postoperative decline of M371 levels have not yet dropped to the normal range despite complete eradication of GCT cells by orchiectomy. In all, postoperatively elevated M371 levels may first arise from large tumours with delayed decay of serum levels and secondly, from premature blood sampling. A novel information of this study is the strong association between preoperative M371 levels and LV1 as shown in the ROC analysis that revealed an AUC of 0.732 for predicting the LV1 status by elevated M371 levels. No clear biological explanation for this relationship is at hand but it is rational to assume that neoplastic cells with direct communication to the vascular system (LV1) may drain their cellular products, e.g. microRNAs directly into the circulation, thus, causing higher M371 levels in these cases. This postulated pathway mirrors the observation of higher M371 levels in testicular vein blood than in the peripheral circulation [29]. A secondary factor causing higher preoperative M371 levels in patients with LV1 is the fact that lymphovascular invasion is more prevalent in larger tumours (Fig. 4). Thus, higher M371 levels in LV1 patients may relate to biological characteristics of vascular drainage and tumour bulk.

We also observed a significant association of preoperative M371 levels with the presence of > 50% EC in the primary. Clearly, the association of preoperative M371 levels with these two recognised risk factors needs to be further evaluated. It appears conceivable that M371 levels above a certain cut-off might quite accurately mirror biological features of the primary tumour including the LV1 status. As M371 levels are measurable by standardised laboratory techniques such measurements could represent a more robust risk marker for aggressive disease than observer-dependent histological markers. However, at present, the clinical significance of the association of preoperative M371 levels with histological risk factors remains elusive.

Another noteworthy finding is the inverse association of teratoma component in the primary with both the presence of lymphovascular invasion (LV1) and the presence of > 50% EC. This finding may point to the lower propensity of teratoma to metastasize and the result is consistent with results of clinical studies that reported lower incidences of relapses in NS CS1 patients who had teratoma components in their primary tumours [30].

In aggregate, we identified several histopathological features of the primary tumour that influence the preoperative M371 serum level. More evidence for this conclusion comes from the multiple regression model that revealed tumour size, > 50% EC, teratoma component, and the LV status to be significant predictors of preoperative M371 levels. According to this model, almost 24% of the variation of preoperative M371 expression (*R*^2^ = 0.239) is related to these factors.

The major limitation of this study is the lack of follow-up data of the patients included. We used the LV1 status as surrogate marker for occult metastases but this marker has a known low sensitivity. Another limitation relates to the lack of information about the intervals of postoperative blood sampling from surgery. The lack of a central pathological review is clearly a weakness since the endpoints of our study relate to histopathological factors. Thus, inconsistencies regarding histopathological assessments of primary tumours cannot entirely be excluded. Also, exact quantification of the amount of embryonal carcinoma in the primary was not possible.

Strengths of the investigation may relate to the prospective multicentric enrolment of patients with minimising selection bias and also to the completeness of data sets in the majority of cases.

## Conclusions

Against expectation, there is no association of postoperatively elevated M371 levels with the LV status. However, the hypothesis is offered that postoperatively elevated M371 levels may for the most part relate to premature blood sample acquisition after orchiectomy and delayed decay of M371 serum levels in large primary tumours. This assumption is based on the association of the velocity of decay of M371 serum levels with primary tumour size. However, it must also be considered that the M371 test with its present methodological features could possibly be too insensitive to detect microscopic foci of germ cell cancer. Noteworthy, preoperative M371 levels showed a remarkable association with the presence of both risk factors, LV1 and > 50% EC, and this relationship clearly deserves further evaluation.

## Abbreviations

AFP: Alpha fetoprotein
bHCG: Beta human chorionic gonadotropin
CS: Clinical stage
EC: Embryonal carcinoma
GCT: Germ cell tumours
IQR: Interquartile ranges
LDH: Lactate dehydrogenase
LV: Lymphovascular invasion
miRNA: MicroRNA
M371: MicroRNA-371a-3p
NS: Nonseminomatous
pT: Pathological stage
RLND: Retroperitoneal lymph node dissection
ROC: Receiver operating characteristics
RQ: Relative quantity
SPSS: Statistical package for the social sciences
ULN: Upper limit of norm
